# Exploring the Relationship Between Childbirth Expectations and Fear Among Primigravida Women in Saudi Arabia

**DOI:** 10.7759/cureus.49337

**Published:** 2023-11-24

**Authors:** Fatimah D Albalawi, Wafaa A Faheem, Hala Thabet, Hanan Daghash

**Affiliations:** 1 Faculty of Nursing, King Abdulaziz University, Jeddah, SAU; 2 Training and Academic Affairs, Ministry of Health, Tabuk, SAU; 3 Health Research, Health Technology, Tabuk, SAU

**Keywords:** wijma delivery expectancy/experience questionnaire, saudi arabia, expectations, primigravida, fear of childbirth

## Abstract

Background

Childbirth is a significant life event that is accompanied by fear, particularly among primigravida mothers. However, little is known about the expectations and fears of childbirth of primigravida women in Saudi Arabia.

Aim

This study aimed to explore expectations and fears of childbirth among primigravidas.

Methods

An exploratory, descriptive, cross-sectional study involving 369 primigravidas was conducted at antenatal outpatient clinics at the Maternity and Children’s Hospital in Tabuk, Saudi Arabia.

Results

In the current study, most participants were aged 25-34 years 204 (55.3%), married 355 (96.2%), and had secondary or higher education 279 (75.6%). A majority of participants 265 (71.8%) reported financial difficulties and 244 unplanned pregnancies (66.1%), while most were pregnant beyond 30 weeks 254 (66.4%). The results showed that the mean total score on the Wijma Delivery Expectancy Questionnaire (W-DEQ) fear of childbirth scale was 57.56, indicating moderate levels of fear on average among primigravida women, with the highest subscale score for the moment of birth. The mean total score on the Childbirth Expectations Questionnaire (CEQ) childbirth expectations scale was 108.15, also indicating moderate expectations on average, with other significant expectations having the highest subscale score. A significant negative correlation was found between the fear and expectations scales (r=-0.775, p<0.001). Sociodemographic factors such as older age, higher education, income, employment, and planned pregnancy were associated with higher expectations and lower fear, whereas younger age, lower education, income, employment, unplanned pregnancy, and medical issues were associated with higher fear.

Conclusions

The findings of this study provide valuable insights into the expectations and fears of childbirth of primigravida women in Saudi Arabia. The results could inform healthcare providers and policymakers about the predictors of fear and effective interventions to reduce fear and improve birth experience in primiparous women.

## Introduction

Childbirth is a profoundly transforming life event for women. Although often joyful, this period can be marked by significant fear and uncertainty, particularly among first-time mothers and primigravida women. Two pivotal psychological factors shape primigravida women's experience with childbirth, including expectations of childbirth and fear of childbirth (FOC) [1-3].

The expectations that a woman has about childbirth before or during pregnancy play a crucial role in shaping her experience and behavior during the birthing process [4]. Most women have expectations or plans for how they hope for labor and delivery [5]. Evidence suggests that childbirth expectations are influenced by previous experiences [6]. The mismatch between birth expectations and birth experiences has been found to be associated with birth satisfaction and can increase the risk of developing postnatal post-traumatic stress disorder (PTSD) [5,6].

Research by Conrad and Trachtenberg (2023) has shown that women have relatively low expectations of childbirth for coping with labor pain and express concerns about the severity of pain [7]. Diverse factors influence childbirth expectations among primigravidas, including birth stories, healthcare providers, and the physical environment [8]. These findings suggest that it is important for nurses and midwives to be aware of women's expectations and to provide appropriate support. Therefore, measuring birth expectations during pregnancy is more rigorous than measuring birth expectations after birth to obtain an accurate understanding of what childbirth will be like.

FOC has emerged as a key concern among primigravida women. FOC is a complex and multidimensional construct, and recent research has identified 10 key elements underpinning the FOC construct: “fear of being abandoned and alone, fear of harm to the women themselves or the baby, and fear of not being able to cope with pain” [9]. This fear can be classified on a continuum ranging from almost no fear to extreme fear [10]. FOC is sometimes attributed to fear of the unknown, but a variety of other factors may also affect it, including nulliparity, younger age, lack of social support, unplanned pregnancy, history of complications, low self-efficacy, and sociodemographic factors [11,12].

The FOC has emerged as a prevalent concern among primigravida women in research spanning diverse global contexts. Studies have reported a FOC prevalence between 10% and 45% in primigravida populations [11,13,14]. Multifactorial factors have been identified, including anxiety, lack of support, previous birth experiences, pregnancy risk status, socioeconomic factors, and cultural norms [11,15]. FOC has been associated with adverse outcomes, such as augmented pain relief needs, emergency cesarean sections, and dissatisfaction with birth experience [16,17].

Mitigating FOC and aligning childbirth expectations with reality among primigravida women is crucial for optimizing the birth experience and maternal well-being. Targeted interventions encompassing emotional support, childbirth education, and woman-centered care models have shown promise in addressing FOC [18]. However, few studies have elucidated the intricate relationship between childbirth expectations and FOC in primigravida women. Given the rising FOC-driven cesarean section rates, this represents a significant knowledge gap, especially within the Saudi context [19].

Therefore, this study aimed to address this gap by exploring FOC levels and childbirth expectations among primigravida women in Saudi Arabia, analyzing the associations between related factors, and evaluating the interrelationships between childbirth expectations and FOC.

## Materials and methods

Research design

The research method chosen for this study was an exploratory, descriptive, cross-sectional design.

Sampling

Convenience sampling was used to recruit primigravida women aged 18-45 years old who were willing to participate. A total of 369 questionnaires were collected. The inclusion criteria for participation in the study were primigravide and Arabic speaking. Women with a history of psychiatric disorders were excluded from this study.

Data collection tools

The self-administered questionnaire consisted of three sections. The first section focused on sociodemographic data and obstetric history. The second section included the Childbirth Expectations Questionnaire (CEQ), a reliable and valid instrument used to assess expectations of nursing support, other significant support, pain/coping ability, need for medical intervention, and comfort/control during labor and delivery. The CEQ is a 35-item self-report instrument consisting of five subscales with each item rated on a 5-point Likert scale. The negative items were reversed and the responses to all items were summed to obtain a total scale score ranging from 35 to 175. The internal consistency of the full scale was acceptable (α = .82) [20].

The third section included the Wijma Delivery Expectancy Questionnaire (W-DEQ), a self-administered instrument that assesses the FOC. The W-DEQ Version A (WDEQ-A) is a reliable and valid tool consisting of 33 items, each with four possible responses on a 6-point scale ranging from "not at all" to "extremely." A high total score indicates a high level of fear, with a cut-off point of 85. Scores of 85 or higher indicated clinical fear, whereas scores of 37 or lower indicated mild fear. Scores between 38 and 65 indicated moderate fear and scores between 66 and 84 indicated intense fear. The WDEQ-A has a good internal consistency, with a Cronbach alpha coefficient of 0.87 [10].

The questionnaire was developed in English and translated into Arabic according to the standard forward-backward procedure. Three nursing experts reviewed the content validity of the questionnaire. We tested its reliability on 30 primigravidas with characteristics similar to those of the target participants in a pilot study. Based on the results, Cronbach's alpha coefficients for the CEQ and W-DEQ versions A were 0.723 and 0.932, respectively. In terms of internal consistency, all coefficients exceeded 0.70, making the questionnaires deemed reliable.

Data analysis

Data were analyzed using descriptive and inferential statistics. Descriptive statistics were used to calculate the mean and standard deviation for continuous data and frequency and percentages for categorical data. Inferential statistics were also carried out, and analysis of variance (ANOVA) and t-tests were performed to determine the association between mothers' characteristics and their level of fear and expectations of childbirth. Pearson’s correlation analysis was conducted to examine the relationship between expectations and FOC among primigravida women. All statistical analyses were performed using Statistical Package for the Social Sciences (IBM SPSS Statistics for Windows, IBM Corp., Version 26.0, Armonk, NY) with a significance level of α = .05.

Ethical considerations

The Institutional Review Board of the General Director of Health Affairs in Tabuk City, Saudi Arabia (with registration number H-07-TU-077 and approval date of 31 January 2023) granted ethical approval for this study.

## Results

Sociodemographic characteristics of primigravida women

Table 1 indicates that more than half of the 204 participants (55.3%) were between the ages of 25 and 34. A total of 355 (96.2%) of the participants were married. A significant proportion of 151 (40.9%) had completed secondary school, and 128 (34.7%) had earned a bachelor's degree. Furthermore, a considerable number mentioned that their incomes barely met their expenses 265 (71.8%). The majority of participants were over 31 weeks pregnant, with 132 (35.8%) in the 36th to 40th gestational week, 113 with 30.6% in the 31st to 35th week, and 124 with 33.6% in the 27th to 30th week. There were two-thirds of participants who reported unplanned pregnancies 244 (66.1%), no medical conditions 231 (62.6%), or being afraid of medical intervention 306 (82.9%).

**Table 1:** Descriptive Sociodemographic

Demographic Characteristic	Number	Percentage
Age		
17-24	135	36.6%
25-34	204	55.3%
35-45	30	8.1%
Marital status		
Married	355	96.2%
Divorced	13	3.5%
Widow	1	0.3%
Occupational status		
Employed	74	20.1%
Housewife	247	66.9%
Self-employed	5	1.4%
Student	43	11.7%
Educational status		
No formal education	13	3.5%
Primary education	77	20.9%
Secondary education	151	40.9%
College and above	128	34.7%
Income level		
Enough	93	25.2%
Barely enough	265	71.8%
More than enough	11	3.0%

Reproductive history of primigravida women

Table 2 represents the reproductive history of primigravida women.

**Table 2:** Descriptive Reproductive History

Obstetric History	Count	Percentage
Weeks of gestational		
27-30	124	33.6%
31-35	113	30.6%
36-40	132	35.8%
Is this current planned pregnancy?		
Yes	125	33.9%
No	244	66.1%
Do you have medical problems during the current pregnancy?		
Yes	138	37.4%
No	231	62.6%
Do you fear having medical intervention during labour?		
Yes	306	82.9%
No	63	17.1%
Is your husband supporting you during pregnancy?		
Yes	329	89.2%
No	40	10.8%
How many times do you visit the antenatal care centre?		
None	6	1.6%
1-2 times	165	44.7%
More than 3 times	198	53.7%
Are you carrying a single foetus or twins?		
Single fetus	363	98.4%
Twins	6	1.6%

Level of childbirth expectation among primigravida women

Table 3 presents the descriptive statistics of the CEQ subscales. The results revealed that the scores for expectations of nursing support were 3.30, pain coping expectations were 2.88, and intervention expectations were 2.95 and 3.36, respectively. The total score for the CEQ indicated that participants had moderate expectations of childbirth (M=108.15, SD=10.04).

**Table 3:** Descriptive Statistics of Childbirth Expectations (N=369)

Subscales of Childbirth Expectations	Mean	Standard Deviation	Maximum	Minimum
Nursing Support	3.30	.43	4.50	2.00
Pain Coping	2.88	.52	4.64	1.55
Intervention	2.95	.35	4.11	1.44
significant Other	3.36	.56	5.00	1.86
Total score of Childbirth Expectation	108.15	10.04	155.00	79.00

Level of FOC among primigravida

In Table 4, the highest mean scores were recorded for the subscales of the moment of birth and social isolation at 2.84 and 2.75, respectively. The total mean score for Version A was 57.56 (SD = 20.55), and the participants experienced a moderate level of apprehension toward childbirth.

**Table 4:** Descriptive Statistics for Subscales of (W-DEQ) Version A (N=369) W-DEQ: Wijma Delivery Expectancy Questionnaire

Subscales of (W-DEQ) Version A	Mean	Standard Deviation	Maximum	Minimum
Social isolation	2.75	.87	5.50	.50
Lack of positive emotions	2.69	.78	5.45	.45
Moment of birth	2.84	.87	5.50	.50
Negative emotions	2.57	.76	5.25	.25
Total Score of (W-DEQ) version A	57.56	20.55	141.00	.00

Correlation between childbirth expectations and FOC

The results of the study revealed a highly statistically significant negative correlation between the W-DEQ and CEQ (r = -0.775, p < 0.001), indicating that an increase in FOC was accompanied by a corresponding decrease in childbirth expectations. These findings suggest a strong association between decreased fear levels and increased childbirth expectations among primigravidas. Table 5 presents the results.

**Table 5:** Correlation between Childbirth Expectations and Fear of Childbirth ** The correlation is significant at the 0.001 level (2-tailed). N = sample size

		Childbirth Expectations
Fear of Childbirth	Pearson Correlation	-.775^**^
	Sig. (2-tailed)	0.001
	N	369

Relationship between primigravida characteristics and expectations of childbirth

The results of the ANOVA and t-test showed differences in expectations among the women with different characteristics. Table 6 presents the variations in expectations based on factors such as age, occupation, education level, and income. Women between the ages of 35-45 had higher childbirth expectations. Self-employed women had higher expectations than employed women, housewives, or students. Women with college or higher education had higher expectations than those without formal, primary, or secondary education. Women with more than a sufficient income had higher expectations than those with sufficient or insufficient income. Women with a planned current pregnancy had higher expectations than those with an unplanned pregnancy, and those with a fear of medical intervention during labor had lower expectations than those without such fear. Women with medical problems during pregnancy have lower expectations than those without such problems. However, carrying single or twin fetuses did not significantly affect childbirth expectations.

**Table 6:** Association between Mother Characteristics and Childbirth Expectations (N=369)

Mother Characteristics	Mean	Standard Deviation	T/F	P
Age category			13.572	.001
17-24	104.90	4.80		
25-34	109.60	11.16		
35-45	113.00	14.60		
Marital status			1.723	.180
Married	108.34	10.15		
Divorced	103.77	5.23		
Widow	99.00	.		
Occupational status			40.461	.001
Employed	117.59	15.39		
House wife	105.45	5.95		
Self-employed	119.80	15.51		
Student	106.09	4.57		
Educational status			31.681	.001
No formal education	101.08	5.24		
Primary education	105.12	5.06		
Secondary education	105.08	3.97		
College and above	114.33	13.98		
Income level			64.664	.001
Enough	115.85	14.14		
Barely enough	104.97	4.62		
More than enough	119.82	18.39		
Weeks of gestational			.586	.557
27-30	107.90	9.14		
31-35	107.58	9.22		
36-40	108.89	11.46		
Is this current planned pregnancy?			-9.315	.001
Yes	114.34	14.07		
No	104.99	4.68		
Do you have medical problems during the current pregnancy?			-2.113	.035
Yes	106.73	5.60		
No	109.00	11.86		
Do you fear having medical intervention during labour?			-9.397	.001
Yes	106.15	6.13		
No	117.89	17.25		
Are you husband support you during pregnancy?			3.193	.001
Yes	108.73	10.28		
No	103.43	6.13		
How many times have you visited the antenatal care center?			7.623	.001
None	109.17	10.83		
1-2 times	105.93	6.37		
More than 3 times	109.98	12.01		
Are you carrying a single fetus or twins?			-.617	.537
Single fetus	108.11	10.12		
Twins	110.67	2.58		

Relationship between demographic characteristics and FOC

According to the ANOVA, there were significant differences in the level of FOC among the primigravidas (see Table 7). Differences were observed across age groups, occupational status, education level, and income level. Primigravid younger women with a lower education, lower income, and housewives tend to have a higher level of FOC. According to the t-test, there was a significant difference in FOC between primigravid women with planned and unplanned pregnancies as well as between those who were concerned about medical intervention. Furthermore, primigravida women who received support from their husbands were significantly less afraid of childbirth than those who did not receive support. In addition, primigravida women with medical problems during pregnancy are more likely to worry about childbirth. Primigravida pregnancies with a single fetus and those with twins reported similar levels of FOC.

**Table 7:** Association between Mother Characteristics and Fear of Childbirth (N=369)

Mother Characteristics	Mean	Standard Deviation	t/F	P
Age category			13.725	.001
17-24	114.49	16.32		
25-34	103.27	27.85		
35-45	93.37	25.59		
Marital status			.476	.622
Married	106.33	25.18		
Divorced	112.38	15.74		
Widow	118.00	.		
Occupational status			36.835	.001
Employed	83.57	34.70		
Housewife	113.28	17.74		
Self-employed	85.60	30.98		
Student	110.07	11.87		
Educational status			27.623	.001
No formal education	125.92	21.10		
Primary education	114.23	17.67		
Secondary education	113.15	14.97		
College and above	92.24	31.14		
Income level			61.289	.001
Enough	87.04	30.56		
Barely enough	114.37	16.37		
More than enough	83.91	37.68		
Weeks of gestational			1.359	.258
27-30	107.54	24.25		
31-35	108.76	20.09		
36-40	103.79	28.82		
Is this a planned current pregnancy?			-9.315	.001
Yes	91.38	32.19		
No	114.35	15.18		
Do you have medical problems during the current pregnancy?			3.620	.001
Yes	112.54	18.74		
No	103.00	27.35		
Do you have fear of having medical intervention during labour?			9.753	.001
Yes	111.69	17.42		
No	81.71	37.80		
Is your husband support you during pregnancy?			-4.261	.001
Yes	104.69	24.65		
No	122.05	21.52		
How many times have you visited the antenatal care center?			5.408	.005
None	109.67	7.89		
1-2 times	111.15	21.72		
More than 3 times	102.66	27.02		
Are you carrying a single fetus or twins?			1.281	.201
Single fetus	106.79	24.74		
Twins	93.67	32.98		

## Discussion

The specific objectives of this study were to explore childbirth expectations, examine the level of FOC and associations between maternal characteristics and expectations/fear, and analyze the relationship between expectations and FOC among primigravida women. In line with these objectives, our findings offer insight into these aspects within the scope of this study.

Sociodemographic and reproductive characteristics of the study sample

The sample was primarily comprised married women aged 25-34 years, indicating that they were in the typical childbearing age range. Most had completed secondary education and were engaged in household duties. However, most studies reported that their income was sufficient only for expenses, highlighting financial considerations.

Reproductive characteristics revealed that most participants had late-stage pregnancies, with over one-third between 36 and 40 weeks of gestation. A substantial proportion of women had unplanned pregnancies, medical issues during pregnancy, and fear of medical interventions. However, most patients receive spousal support and adequate antenatal care.

The childbirth expectations among primigravida

Primigravida women had moderately positive expectations of childbirth, particularly regarding nursing support. This observation is consistent with previous studies that emphasized the importance of supportive relationships and care providers in shaping positive childbirth expectations [21,22]. However, expectations regarding pain management and medical intervention are moderate or neutral. This finding concurs with qualitative research highlighting how fear related to unpredictability, medical procedures, and pain can significantly influence childbirth expectations [23,8].

The association between maternal characteristics and childbirth expectations

Maternal age significantly influenced childbirth expectations, with women aged 35-45 displaying higher expectations than younger groups. This aligns with Conrad and Trachtenberg's (2023) research on personality traits and fear [7], which suggests that older women may have different traits or experiences that shape their outlook.

Occupational status also played an important role. Self-employed women had markedly higher expectations than employed women, housewives, and students did. These findings on care environment concerns imply that perceived control may increase expectations [8]. Interventions to enhance the perceptions of control may be beneficial. Additionally, higher education level was associated with greater expectations, consistent with a previous study [23]. This indicates that educated women may be better equipped to make informed decisions, thus contributing to higher expectations. Providers should consider educational disparities when addressing their expectations and autonomy. Income level also significantly shapes expectations. Women with surplus income had higher expectations than those with sufficient or insufficient income, which is consistent with previous research on financial status [24]. Alleviating cost concerns through financial literacy initiatives may improve this outlook.

Furthermore, planned pregnancy has also emerged as an influential factor, with planned pregnancies linked to higher expectations [23]. This finding suggests that perceived control over timing promotes positivity. Support for women with unplanned pregnancies can help to align their expectations. Also, fear of medical interventions predicted lower expectations, finding previously in research that addressed fear of enhancing experiences [7]. Comprehensive information and support are vital for reducing fear.

Additionally, husbands’ support during pregnancy is associated with higher expectations, consistent with previous findings [22]. Social support is a key to expectations and well-being. Engaging partners can optimize their experiences. Finally, medical issues during pregnancy predicted lower expectations, which is consistent with the findings of previous studies [5,25]. Prioritizing care for such women is crucial.

The FOC among primigravida

This study sheds light on the level of FOC among primigravida women and the various factors that influence this fear. The prevalence of moderate fear identified in our study is consistent with previous quantitative research that reported moderate fear among primigravida women [14,26,27]. These findings underscore the need for a comprehensive understanding of this issue and tailored interventions to address it effectively.

Association between maternal characteristics and FOC

Several factors associated with higher FOC were aligned with previous research, including sociodemographic characteristics and obstetric history [11-13]. A key finding was the association between maternal age and the FOC. Women aged 17-24 exhibited higher fear than those aged 25-34 and 35-45, similar to the findings [28]. This finding highlights the need for age-specific interventions to address developmental concerns.

Fear levels also differed according to occupational status. Housewives had significantly higher fear than employed women, self-employed women, or students, which is consistent with a previous study [16]. This demonstrates the role of occupational factors in shaping fear, emphasizing the need for tailored support based on work status. Additionally, lower educational attainment was associated with greater fear. Women without formal education showed higher fear than those with primary, secondary, or higher education, which is consistent with other findings [12]. This underscores the value of educational initiatives in addressing knowledge gaps and concerns based on education levels. Socioeconomic aspects also contribute to fear, with women of insufficient income exhibiting higher fear than those with sufficient income, which is consistent with a previous study [29]. Comprehensive interventions are vital for addressing financial concerns among low-income primigravidas.

Several obstetric factors were also found to be predictive. Planned pregnancies had lower fear levels than unplanned pregnancies, which is consistent with previous findings, highlighting the importance of pregnancy planning [15]. Moreover, fear of medical interventions predicted a greater FOC, consistent with a previous study [1]. Offering appropriate counseling for interventions is crucial. This study also identified that women receiving support from their husbands during pregnancy had lower levels of FOC, highlighting the significance of spousal involvement in reducing fear among primigravida women [30]. These findings collectively contribute to a deeper understanding of the multifaceted factors influencing FOC among primigravida women, and provide insights for targeted interventions in antenatal care.

The relationship between expectations of childbirth and FOC among primigravida women

This study examines the relationship between expectations and FOC among first-time mothers. A significant negative correlation was observed, indicating that higher FOC was associated with lower childbirth expectations. This finding is consistent with those of previous studies [1-3]. Women with higher levels of fear tend to have lower expectations as they may anticipate a negative outcome and experience anxiety and stress. Therefore, it is crucial to provide comprehensive support and interventions to address fear and to promote positive expectations among first-time mothers.

Implications for practice

Childbirth education programs should provide realistic information about labor, delivery, and possible interventions to help align the expectations of primigravida women, addressing critical knowledge gaps and misconceptions. Antenatal care should include screening for FOC using validated tools, with referral for counseling and support interventions for women with high fear levels. Counseling and emotional support from nurses are also critical to help primigravidas verbalize and work through their childbirth concerns using active listening and validation techniques. Involving partners in antenatal education and counseling for social support and spousal participation should be encouraged. Customized childbirth preparation programs tailored to women's age, education, income, pregnancy planning, and other attributes may help address specific concerns. Especially for those with a high fear of medical interventions, additional information and counseling on necessity and safety are key, along with informed consent and shared decision-making.

Limitations and future work

This study has limitations due to its cross-sectional design, which prevents determining causal relationships between the factors examined and expectations or FOC. The self-reported nature of the measures also introduces the possibility of recall or response bias, affecting the results. Although validated tools were used, self-reported data may not fully capture constructs of interest. Addressing these limitations in future studies is therefore crucial. A longitudinal approach would allow the exploration of causal relationships, whereas mixed-method approaches combining quantitative and qualitative data would provide a more comprehensive understanding. Expanding the sample size, including diverse populations, and exploring cultural and regional differences would enhance future research.

## Conclusions

This study sheds light on the multifaceted factors influencing childbirth expectations and fear among primigravida women. It underscores the significance of age, occupation, education, income, pregnancy circumstances, medical issues, partner support, and attendance in antenatal care in determining these aspects. Nurses should use this knowledge to tailor their services and provide comprehensive support and interventions to optimize childbirth expectations and alleviate fear, ultimately enhancing maternal well-being.

## References

[1] Rúger-Navarrete A, Vázquez-Lara JM, Antúnez-Calvente I (2023). Antenatal fear of childbirth as a risk factor for a bad childbirth experience. Healthcare (Basel).

[2] Preis H, Lobel M, Benyamini Y (2023). Between expectancy and experience: testing a model of childbirth satisfaction. J Reprod Infant Psychol.

[3] Raudasoja M, Sorkkila M, Vehviläinen-Julkunen K, Tolvanen A, Aunola K (2022). The role of self-esteem on fear of childbirth and birth experience. J Reprod Infant Psychol.

[4] Demirel G, Kaya N, Evcili F (2022). The relationship between women's perception of support and control during childbirth on fear of birth and mother's satisfaction. J Obstet Gynaecol.

[5] Webb R, Ayers S, Bogaerts A (2021). When birth is not as expected: a systematic review of the impact of a mismatch between expectations and experiences. BMC Pregnancy Childbirth.

[6] Korukcu O, Bulut O, Kukulu K (2019). From experiences to expectations: a quantitative study on the fear of childbirth among multigravida women. Arch Psychiatr Nurs.

[7] Conrad MS, Trachtenberg E (2023). Personality traits, childbirth expectations, and childbirth experiences: a prospective study. J Reprod Infant Psychol.

[8] Turan Z, Suveren Y, Vural G (2022). A qualitative study on the expectations and experiences of mothers during the childbirth process in Western Anatolia, Turkey. Women Health.

[9] Slade P, Balling K, Sheen K, Houghton G (2019). Establishing a valid construct of fear of childbirth: findings from in-depth interviews with women and midwives. BMC Pregnancy Childbirth.

[10] Wijma K, Wijma B, Zar M (1998). Psychometric aspects of the W-DEQ; a new questionnaire for the measurement of fear of childbirth. J Psychosom Obstet Gynaecol.

[11] Dencker A, Nilsson C, Begley C (2019). Causes and outcomes in studies of fear of childbirth: a systematic review. Women Birth.

[12] Gökçe İsbir G, Serçekuş P, Yenal K (2022). The prevalence and associated factors of fear of childbirth among Turkish pregnant women. J Reprod Infant Psychol.

[13] Dereje A, Dheresa M, Desalew A, Tura AK (2023). Fear of childbirth among pregnant women in Eastern Ethiopia: a community-based study. Midwifery.

[14] Khosravi P, Pirdadeh Beiranvand S, Beiranvand B, Khalesi ZB (2022). Relationship between primigravid women's awareness, attitude, fear of childbirth, and mode of delivery preference. Eur J Obstet Gynecol Reprod Biol X.

[15] Shakarami A, Mirghafourvand M, Abdolalipour S, Jafarabadi MA, Iravani M (2021). Comparison of fear, anxiety and self-efficacy of childbirth among primiparous and multiparous women. BMC Pregnancy Childbirth.

[16] Serçekuş P, Vardar O, Özkan S (2020). Fear of childbirth among pregnant women and their partners in Turkey. Sex Reprod Healthc.

[17] Stoll KH, Hauck YL, Downe S (2017). Preference for cesarean section in young nulligravid women in eight OECD countries and implications for reproductive health education. Reprod Health.

[18] Wigert H, Nilsson C, Dencker A (2020). Women's experiences of fear of childbirth: a metasynthesis of qualitative studies. Int J Qual Stud Health Well-being.

[19] Ahmed AE, Mohammad RS (2018). Cesarean sections. Associated factors and frequency at King Abdulaziz Medical City in the Central Region of the Kingdom of Saudi Arabia. Saudi Med J.

[20] Gupton A, Beaton J, Sloan J, Bramadat I (1991). The development of a scale to measure childbirth expectations. Can J Nurs Res.

[21] Murray-Davis B, Grenier LN, Mattison C (2023). Mediating expectations and experiences that influence birth experiences in Canada's first Alongside Midwifery Unit. Birth.

[22] McLeish J, Harvey M, Redshaw M, Alderdice F (2020). "Reassurance that you're doing okay, or guidance if you're not": a qualitative descriptive study of pregnant first time mothers' expectations and information needs about postnatal care in England. Midwifery.

[23] Arik RM, Parada CM, Tonete VL, Sleutjes FC (2019). Perceptions and expectations of pregnant women about the type of birth. Rev Bras Enferm.

[24] Gwacham-Anisiobi UC, Banke-Thomas A (2020). There is no ideal place, but it is best to deliver in a hospital: expectations and experiences of health facility-based childbirth in Imo State, Nigeria. Pan Afr Med J.

[25] Schaal NK, Hagenbeck C, Helbig M, Wulff V, Märthesheimer S, Fehm T, Hepp P (2023). The influence of being pregnant during the COVID-19 pandemic on birth expectations and antenatal bonding. J Reprod Infant Psychol.

[26] Hendrix YM, Baas MA, Vanhommerig JW, de Jongh A, Van Pampus MG (2022). Fear of childbirth in nulliparous women. Front Psychol.

[27] Johnson AR, Kumar MG, Jacob R, Jessie MA, Mary F, Agrawal T, Raman V (2019). Fear of childbirth among pregnant women availing antenatal services in a maternity hospital in rural Karnataka. Indian J Psychol Med.

[28] Huang J, Huang J, Li Y, Liao B (2021). The prevalence and predictors of fear of childbirth among pregnant Chinese women: a hierarchical regression analysis. BMC Pregnancy Childbirth.

[29] Qiu L, Sun N, Shi X, Zhao Y, Feng L, Gong Y, Yin X (2020). Fear of childbirth in nulliparous women: a cross-sectional multicentre study in China. Women Birth.

[30] Buldum A, Güner Emül T (2021). The fear of childbirth and social support in adolescent pregnancy. J Pediatr Adolesc Gynecol.

